# Evaluation of the clinical efficacy of ultra‐fast track anesthesia for endoscopic thoracic sympathectomy of palmar hyperhidrosis

**DOI:** 10.1111/jocd.16425

**Published:** 2024-06-19

**Authors:** Wenqing Zhang, Jinglian Lin, Huijuan Zhao, Xuhang Chen, Zhijian Lin, Jian Lin

**Affiliations:** ^1^ Department of Anesthesiology Zhangzhou Affiliated Hospital of Fujian Medical University Fujian China; ^2^ Department of Radiology Zhangzhou Affiliated Hospital of Fujian Medical University Fujian China

**Keywords:** endoscopic thoracic sympathectomy (ETS), enhanced recovery, local infiltration anesthesia, palmar hyperhidrosis, ultra‐fast track anesthesia (UFTA) technique

## Abstract

**Objective:**

In this study, we investigated the safety and practicability of ultra‐fast track anesthesia (UFTA) for endoscopic thoracic sympathectomy (ETS).

**Methods:**

A total of 72 patients with palmar hyperhidrosis undergoing ETS were randomly divided into three groups: the UFTA group (group I), the group undergoing single‐lumen tracheal intubation with local infiltration anesthesia technique (group II), and the group undergoing single‐lumen tracheal intubation with routine anesthesia (group III). Mean arterial pressure (MAP) and heart rate (HR) were recorded for all three groups at the following six time points: Before anesthetics administration (T0), the time of intubating or inserting laryngeal mask airway (T1), the time of incising skin (T2), the time of disconnecting of the right sympathetic nerve (T3), the time of disconnecting of the left sympathetic nerve (T4), the time of withdrawing the tracheal tube or laryngeal mask airway (T5), and the time of transferring the patient to a post‐anesthesia care unit (PACU) (T6). The three groups were compared from the following perspectives: surgery duration; anesthesia recovery duration, that is, the duration from discontinuation of anesthesia to extubating the tracheal tube; the dose of propofol and remifentanil per kilogram body mass per unit time interval (the time at the end of the procedure, which lasted from anesthesia induction to incision suturing); and the visual analog scale (VAS) in the resting state in the PACU.

**Results:**

Based on pairwise comparisons, the average HR and average MAP values of the three groups differed significantly from T2 to T6 (*p* < 0.05). As demonstrated by the correlation analysis between remifentanil and propofol with HR and MAP, the doses of the total amount of remifentanil and propofol were lower, and group I used less remifentanil and propofol than group II. No patient in group I experienced throat discomfort following surgery. Patients in groups II and III experienced a range of postoperative discomfort. The VAS scores of groups I and II were significantly lower than those of group III, with group I lower than group II.

**Conclusion:**

When utilized in ETS, UFTA can provide effective anesthesia for minor traumas. It is safe, effective, and consistent with the enhanced recovery philosophy of fast‐track surgery departments.

## 1 INTRODUCTION

Primary palmar hyperhidrosis is an idiopathic disease characterized by abnormal sweat excretion due to hypersecretion of sweat glands innervated by the thoracic sympathetic nerves.^1^ The benefits of endoscopic thoracic sympathectomy (ETS) include minimal trauma, clear views, targeted locations, satisfactory efficacy, and rapid recovery following surgery. It is the only minimally invasive procedure currently capable of effectively treating palmar hyperhidrosis.^2^ General anesthesia is required when performing ETS. Among the numerous anesthesia techniques described in clinical reports, ultra‐fast track anesthesia (UFTA) was employed in this study using the guidance of enhanced recovery after surgery (ERAS) concept to facilitate minimal invasiveness during both the surgical and anesthesia procedures.^3^ As a result, patients experienced minimal trauma, rapid recovery, a satisfactory anesthetic effect, and few anesthesia‐related complications during their treatment. We compared UFTA and conventional anesthesia techniques to provide a reference for clinical practice.

## 2 DATA AND METHODS

### 2.1 General information

This research was approved by the our hospital's ethics committee (2023LWB224). Between January 2018 and December 2022, we enrolled 72 patients (35 males and 37 females) with palmar hyperhidrosis undergoing ETS at our hospital.

Within this study, heart rate (HR), mean arterial pressure (MAP), and the Visual Analogue Scale (VAS) emerged as pivotal benchmarks for evaluating intraoperative pain levels. Preceding the sample size computation, an initial trial encompassed the selection of four patients per procedure, with meticulous recording of HR and MAP values. Following this, the mean and variance calculations of HR and MAP data from the four patients within each group were meticulously performed, serving as the cornerstone for the subsequent sample size estimations.

The numerical differences of patients' indicators under different anesthesia protocols were compared, so the following formula was used for sample size calculation:

$$n=\varphi^{2}\left(\frac{\sum S_{i}^{2}}{g}\right)/\left[\frac{\sum {\left(X_{i}-\bar{X}\right)}^{2}}{g-1}\right]$$

The g is the number of groups, which is 3 in this study; $X_{i}$ is the mean value of each group, $\;\bar{X}$ is the average of the mean values of the three groups. $S_{i}$ is the standard deviation corresponding to each group. φ is the calculation coefficient corresponding to the different *α* with *β* values;

The specific calculation process is taken. The HR and MAP data at the moment of T3 were used to calculate the above formulas, respectively, and the calculated values for the minimum sample size were 3 (2.901), 14 (13.80). For the actual analysis, a larger sample size of 24 was used to further ensure the credibility of the results.

The patients ranged in age from 13 to 29 years and were classified as American Society of Anesthesiologists (ASA) grade I–II. The patients were divided into three groups based on the anesthesia techniques used: the UFTA group (group I), the group undergoing single‐lumen tracheal intubation combined with local infiltration anesthesia technique (group II), and the group undergoing single‐lumen tracheal intubation and standard anesthesia (group III). There were no statistically significant differences (*p* > 0.05) between the three groups in terms of demographic data.

### 2.2 Anesthesia methods

All patients routinely fasted for 8 h, abstained from drinking water for 2 h, and were not given any anticholinergic medications. Patients were administered oxygen through a nasal catheter at a rate of 3 L/min and their electrocardiogram (ECG), blood pressure (BP), HR, saturation of peripheral oxygen (SpO2), and bilateral palm temperature were monitored after entering the surgery room. After peripheral venous access was established and 15 min prior to induction of anesthesia, patients were intravenously pumped with a standard dose of dexmedetomidine for sedation, namely 0.5 μg/kg (4 μg/mL). The dexmedetomidine pumping was completed within 15 min. Intravenous injections of propofol at 2.0 mg/kg, sufentanil at 0.2 μg/kg, and cisatracurium besilate at 0.1 mg/kg were administered to induce anesthesia. Remifentanil at 0.2 μg/kg/min was concurrently continuously pumped until the incision was sutured at the end of the surgery. For the ventilation of group I, double‐lumen laryngeal mask airways of an appropriate model (3–4#) were inserted into their mouths. For groups II and III, propofol was administered intravenously at a dose of 1.0 mg/kg prior to intubation, and single‐lumen tracheal tubes of the appropriate model (7.0–7.5#) were inserted using a videolaryngoscope. Parameters for breath control were set as oxygen flow between 1.0 and 1.5 L/min, tidal volume (V_T_) between 6 and 8 mL/kg, respiratory rate (RR) between 10 and 15 breaths per minute, and inhalation duration: expiration length (I:E) = 1:2. The patients were positioned in a high Fowler's position with the upper body at 75 degrees, with both arms extending outwards to form a 90° angle with chest and fixed on a frame. The bilateral armpits were exposed, as shown in Figure 1. Maintenance of anesthesia: closed circuit with oxygen flow at 2/L. Trace amounts of propofol, at 2–3 mg/kg/h, and dexmedetomidine, at 0.4 μg/kg/h were continuously pumped into the three groups until the time of disconnecting of bilateral sympathetic nerves. Before the start of the surgery, three additional doses of sufentanil 0.1 μg/kg were administered to the three groups. Thoracoscopy with a minimally invasive single‐port incision has been the most preferable option in our hospital for numerous years. For groups I and II, local infiltration anesthesia using 0.5% ropivacaine was performed at the incision site before skin incision. For group III, infiltration injection was performed with 0.9% sodium chloride in the same doses as in groups I and II. Ventilation was paused before separating the pleura. The threaded tube connection was separated to allow the lung's apex to naturally collapse until the entire field of view was exposed. Subsequently, the threaded tube was connected, and the pressure‐limiting valve was opened. The fresh oxygen flow rate was increased to 6 L/min. If the SpO2 dropped below 90% due to a prolonged apnea duration, high‐frequency and low V_T_ manual transitional ventilation was conducted (without affecting the surgical field exposure), until the SpO2 was restored to above 95%. Concurrently, palm temperature was monitored with the disconnection of the sympathetic nerve, with an increase of above 1°C indicating a successful operation. An 8F indwelling urethral catheter was inserted and connected to the negative pressure suction device. While the pressure‐limiting valve was manually adjusted to fully inflate the lungs, negative pressure was used to suction the gas in the chest cavity. This was continuously implemented until the patient's lungs were completely expanded. Then, the incision was closed and sutured (Figure 2). Anesthesia was discontinued after the completion of lung inflation on the final side of the chest in all three groups. Suctioning was performed to remove the phlegm. An antagonist to the muscle relaxant was administered after the initial respiration was restored. The laryngeal mask airway or endotracheal tube was removed after stabilization of the patient's respiratory and circulatory systems, and the patient was then transferred to the post‐anesthesia care unit (PACU).

**FIGURE 1:**
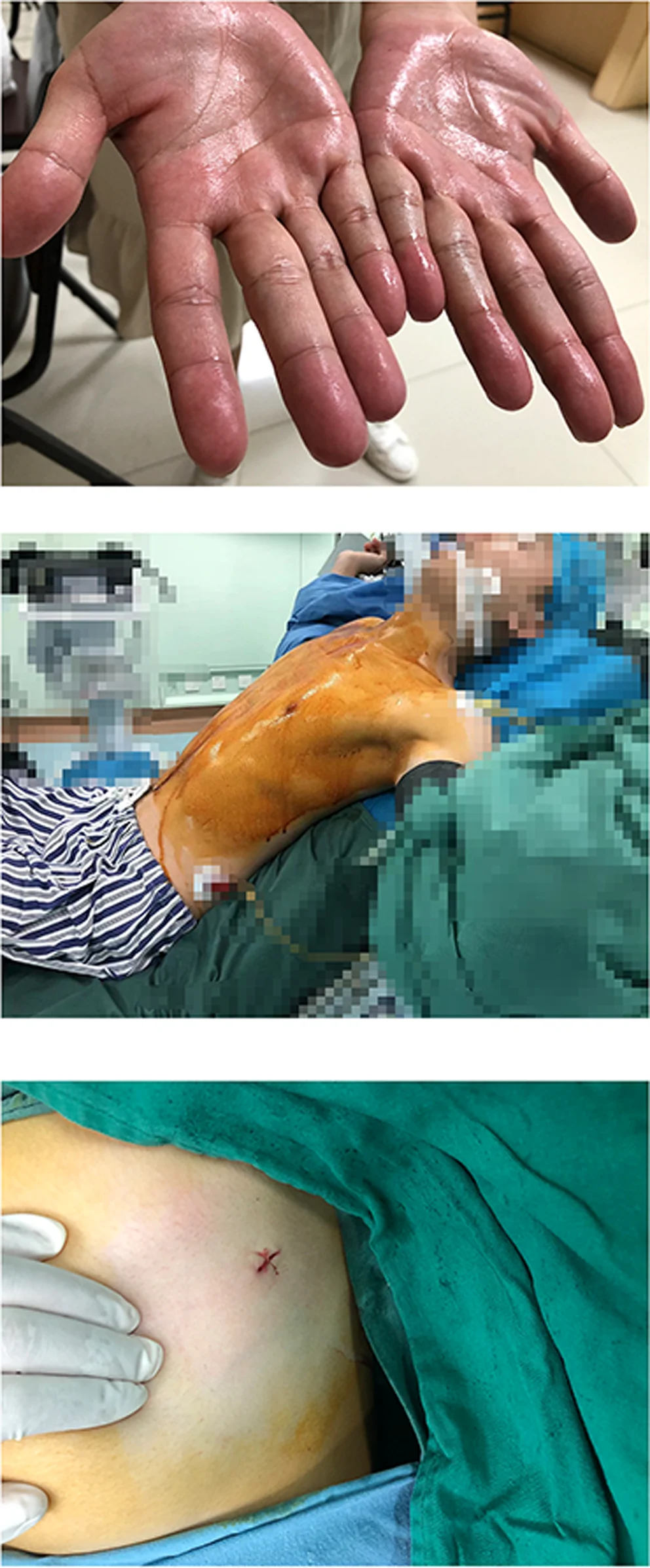
Patient symptoms, surgical body posture, and minimally invasive surgical incision.

**FIGURE 2:**
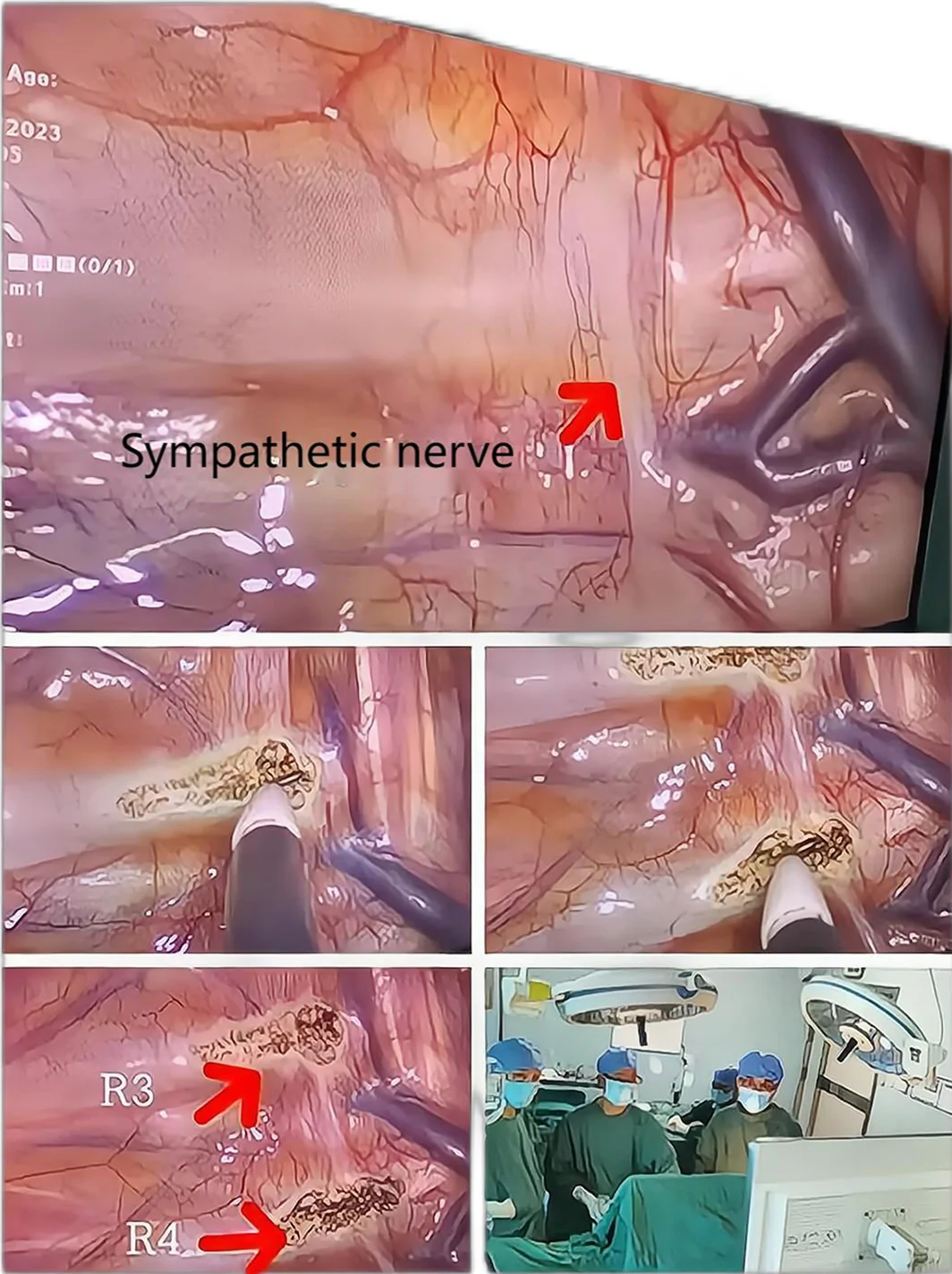
Surgical procedure.

### 2.3 Observational indicators

The UFTA technique was used in the observational group (group I). The control groups were group II—which underwent single‐lumen tracheal intubation combined with local infiltration anesthesia technique, and group III—the group undergoing single‐lumen tracheal intubation and standard anesthesia. (1) MAP and HR were recorded for all three groups at the following six time points: before anesthetics administration (T0), the time of intubating or inserting laryngeal mask airway (T1), the time of incising skin (T2), the time of disconnecting of the right sympathetic nerve (T3), the time of disconnecting of the left sympathetic nerve (T4), the time of withdrawing tracheal tube or laryngeal mask airway (T5), and the time of transferring the patient to the PACU (T6). (2) The following variables were compared and recorded for the three groups: surgery duration; anesthesia recovery duration, that is, the duration from discontinuation of anesthesia to extubation of the tracheal tube; the used doses of propofol and remifentanil per kilogram body mass per unit time interval (the time at the end of the procedure, which lasted from the induction of anesthesia to incision suturing); and the visual analog scale (VAS) score in the resting state in the PACU. (3) The VAS score of each patient in the resting state in the PACU was recorded.

### 2.4 Statistical analysis

SPSS 20.0 software was used for the statistical analysis of data. The enumeration data are represented as the number of cases and corresponding percentages, with the chi‐squared test being used for inter‐group comparisons. The measurement data are expressed as mean ± standard deviation ($\bar{x}$ ± s), with one‐way analysis of variance (ANOVA) used for inter‐group comparisons. The Bonferroni test was used for postprocess verification. Additionally, repeated measures ANOVA analyses were conducted separately based on HR and MAP data. A statistically significant difference was considered when the value of *p* was less than 0.05 (*p* < 0.05).

## 3 RESULTS

1. A total of 72 patients were included in this study, consisting of 33 males and 39 females. All patients underwent a successful operation. Clinical data from the three groups were analyzed, and the average values and standard deviations were calculated for each group. The three groups had no statistically significant difference between age, body mass index (BMI), and surgery duration (*p* > 0.05, Table 1), and the statistically significant differences in anesthesia recovery duration (*p* = 0.022). Based on the postprocess verification using Bonferroni test, the anesthesia recovery duration for group I was significantly lower than group III (*p* = 0.021). There was no statistically significant difference in anesthesia recovery duration between groups I and II (*p* = 0.184) and groups II and III (*p* = 1.000).
2. Comparisons of HR and MAP between the three groups: Pairwise comparisons of HR and MAP average values between the three groups revealed no statistically significant difference at T0 (before anesthetics administration) and T1 (the instant of intubating or inserting laryngeal mask airway) (*p* > 0.05), but displayed statistically significant differences at T2 (the instant of incising skin), T3 (the instant of disconnecting of the right sympathetic nerve), T4 (the instant of disconnecting of the left sympathetic nerve), T5 (the instant of withdrawing tracheal tube or laryngeal mask airway), and T6 (the instant of sending a patient into a PACU) (*p* < 0.05). (Tables 2,3).
3. Analysis of the correlation between HR and MAP and remifentanil and propofol: According to the correlation analysis, MAP was positively correlated with the doses of remifentanil and propofol, with the correlation coefficient *R* ^2^ of 0.543 and 0.366, respectively. HR was positively correlated with the doses of remifentanil and propofol, with the correlation coefficient *R* ^2^ of 0.527 and 0.388, respectively. The doses of remifentanil were more closely correlated with HR and MAP compared to that of propofol. The comparison of propofol doses between the three groups is displayed in Table 4. The least doses of propofol were administered to Group I, while the most doses of propofol were administered to Group III. There were statistically significant differences in propofol doses between the three groups. The comparison of remifentanil doses between the three groups is shown in Table 5. The least doses of remifentanil were administered to Group I, while the most doses of remifentanil were administered to Group III. There were statistically significant differences between the three groups in the doses of remifentanil.
4. Visual analog scale after patients were transferred to the PACU: VAS scores after surgery for groups I, II, and III were 1.08 ± 0.77, 2.45 ± 0.66, and 4.68 ± 0.78, respectively. A comparison of VAS scores between the three groups is displayed in Table 6; there were statistically significant differences between the three groups.
5. This study conducted repeated measures ANOVA analysis based on HR and MAP data. The time points T0–T6 were considered as the repeated measures factor, and the three surgical procedures were considered as the main factor for the multivariate repeated measures ANOVA analysis. For HR data, the assumption of sphericity could not be met based on the Mauchly's test (*p* = 0.000 < 0.05); therefore, Pillai's Trace was chosen as the primary indicator in the multivariate test, yielding a value of 1.647, *F* = 50.573, *p* < 0.001. The repeated measures ANOVA results demonstrated that there was a statistically significant difference in HR values among different surgical procedures when considering the time factor. Similarly, for MAP data, the assumption of sphericity could not be met based on the Mauchly's test (*p* = 0.000 < 0.05), requiring attention to the multivariate test as well. Pillai's Trace was selected as the primary indicator, with a value of 1.483, *F* = 31.057, *p* < 0.001, indicating that there was a statistically significant difference in MAP values among different surgical procedures.

**TABLE 1:** Comparison of baseline data between the three groups.

Item	Group I (*n* = 24)	Group II (*n* = 24)	Group III (*n* = 24)	*p* Value
Age (Years)	19.29 ± 4.12	19.54 ± 4.07	19.79 ± 3.46	0.906
BMI (kg/m^2^)	21.98 ± 2.12	21.58 ± 1.95	21.69 ± 1.82	0.767
Surgery duration (min)	29.75 ± 2.90	29.63 ± 3.84	30.08 ± 2.65	0.875
Anesthesia recovery duration (min)	4.79 ± 1.22	5.33 ± 0.87	5.58 ± 0.83^a^	0.022

*Note*: Group I: a combination of laryngeal mask airway with the UFTA technique. Group II: a combination of single‐lumen tracheal intubation and UFTA. Group III: standard anesthesia with a single‐lumen tracheal intubation. BMI: body mass index. The measurement data were analyzed using one‐way ANOVA, with *p* < 0.05 indicating a statistically significant difference. The three sets of data are the results of mean and standard deviation.

^a^ *p* < 0.05 versus group I.

**TABLE 2:** Inter‐group analysis of HR at various time points.

Item	Group I (n = 24)	Group II (n = 24)	Group III (n = 24)	*p* Value
T0	85.58 ± 6.61	87.33 ± 6.72	84.75 ± 7.91	0.442
T1	61.46 ± 6.41	63.25 ± 3.57	62.33 ± 3.46	0.420
T2	57.46 ± 5.47	63.54 ± 3.16^a^	77.04 ± 3.79^a^, ^b^	<0.001
T3	55.29 ± 4.76	71.3 ± 3.64^a^	81.67 ± 3.66^a^, ^b^	<0.001
T4	54.75 ± 4.83	71.63 ± 3.21^a^	81.96 ± 3.07^a^, ^b^	<0.001
T5	58.42 ± 5.37	77.21 ± 3.87^a^	85.63 ± 2.36^a^, ^b^	<0.001
T6	60.83 ± 5.17	70.83 ± 3.68^a^	75.17 ± 3.56^a^, ^b^	<0.001

*Note*: Inter‐group comparisons were analyzed using one‐way ANOVA. The Bonferroni method was used to conduct the postprocess pairwise comparisons.

^a^ *p* < 0.001 versus group I.

^b^ *p* < 0.001 versus group II.

**TABLE 3:** Inter‐group analysis of MAP at various time points.

Item	Group I (*n* = 24)	Group II (*n* = 24)	Group III (*n* = 24)	*p* Value
T0	85.29 ± 4.84	84.83 ± 4.66	84.04 ± 5.60	0.693
T1	71.17 ± 4.62	70.29 ± 4.06	71.04 ± 5.75	0.797
T2	69.50 ± 4.05	72.21 ± 3.90^a^	80.17 ± 5.01^a^, ^b^	<0.001
T3	69.04 ± 3.11	79.58 ± 4.19^a^	82.5 ± 3.78^a^, ^b^	<0.001
T4	67.96 ± 2.91	79.04 ± 3.45^a^	83.42 ± 2.89^a^, ^b^	<0.001
T5	70.25 ± 2.95	80.92 ± 3.22^a^	86.71 ± 2.93^a^, ^b^	<0.001
T6	64.25 ± 2.61	76.50 ± 2.54^a^	73.58 ± 3.88^a^, ^b^	<0.001

*Note*: Inter‐group comparisons were analyzed using one‐way ANOVA. The Bonferroni method was used to conduct postprocess pairwise comparisons.

^a^ *p* < 0.05 versus group I.

^b^ *p* < 0.05 versus group II.

**TABLE 4:** Pairwise comparisons of propofol doses.

Propofol	Average value	Standard error	*p* Value
Group I versus group II	−24.083	3.119	<0.001
Group I versus group III	−52.208	3.119	<0.001
Group II versus group III	−28.125	3.119	<0.001

**TABLE 5:** Pairwise comparisons of remifentanil doses.

Remifen	Average value	Standard error	*p* Value
Group I versus group II	−11.333	3.564	0.007
Group I versus group III	−207.792	3.564	<0.001
Group II versus group III	−196.458	3.119	<0.001

**TABLE 6:** Pairwise comparisons of VAS scores between the three groups.

VAS	Average value	Standard error	*p* Value
Group I versus group II	−1.375	0.213	<0.001
Group I versus group III	−3.00	0.213	<0.001
Group II versus group III	−1.625	0.213	<0.001

## 4 DISCUSSION

The *Expert Consensus on Minimally Invasive Treatment for Palmar Hyperhidrosis in China* clearly states that ETS is the only effective and long‐lasting minimally invasive method for treating palmar hyperhidrosis.^4^ In recent years, the thoracic surgery department in our hospital has been innovating and accumulating experience with respect to this disorder. Currently, the most common approach used in our hospital is the nonartificial pneumothorax single‐port microincision, in which both the electrocoagulation hook and the 30° endoscope enter the chest cavity through the same incision. This technique provides a better surgical field, accurate positioning, safety and effectiveness, fewer complications, and a concealed and esthetically pleasing incision.^5^ In recent years, experts and scholars have reported that the sympathetic nerve chain clamping surgery can effectively prevent the development of incapacitating compensatory sweating; however, if compensatory sweating occurs during the clamping process, removal of the clamp can effectively reverse the symptoms in patients.^6^ Our hospital has also made significant progress in anesthesia methods in order to better support surgeons and anesthesiologist and properly manage anesthesia. Previously, general anesthesia with double‐lumen tracheal intubation has been the standard procedure. However, it involves expensive consumables and can also lead to anesthetic traumas due to intubation and many postoperative complications. Surgery cases using nontracheal intubation combined with local anesthesia have been previously reported.^7^ Though performing surgery on patients who are awake or sedated^8^ conforms to the ERAS perioperative management concept in thoracic surgery^9^ and benefits patients throughout the perioperative period, it violates the systematic remarks and consensus definitions advocated in the Standardized Endpoints in Perioperative Medicine (StEP) initiative—patient comfort. However, allowing patients to retain autonomous respiration or combining it with artificial pneumothorax to induce lung atelectasis does not provide artificial control over lung ventilation, thereby affecting the surgical field of view.^10^ Though performing surgery using general anesthesia without intubation reduces intubation traumas and cardiovascular responses, excessive sedation and analgesia may result in patients developing respiratory depression and hypoxemia. Therefore, it is imperative to utilize safe and accurate anesthesia methods. Currently, the most prevalent anesthesia method is general anesthesia with single‐lumen tracheal intubation. However, airway and cardiovascular system responses to intubation require better anesthesia management solutions. Glottis ventilation using a double‐lumen laryngeal airway mask is free from complications caused by intubation. It is widely used in clinical practice due to its advantages such as prevention of gastric distension, reflux, and aspiration, better compatibility with the anatomy of the pharynx and larynx, better sealing performance, secure fixation, and low‐frequency displacement.

Endoscopic thoracic sympathectomy, which belongs to fast‐track surgery departments,^11^ is advantageous in minimal invasiveness, fast recovery after surgery, short hospitalization, etc., The ERAS concept advocates for preoperative reduction of patient anxiety, shortening of fasting and water‐drinking time, minimizing surgical trauma, reducing anesthesia medication, and providing effective postoperative pain relief. This study was guided by the ERAS concept and aimed to improve anesthesia methods. The surgery was performed using UFTA technique, which was initially used for off‐pump coronary artery bypass graft (CABG) surgery and is now widely used for anesthesia management in pediatric cardiac surgery and minimally invasive cardiac surgery under thoracoscopes.^12^ Anesthetic analgesics is used in small doses in anesthesia that combines the UFTA technique and laryngeal mask airway. In this case, patients will suffer obvious pain after surgery when they are awake and the tube is removed. Therefore, to achieve safe and effective analgesia during and after surgery, the UFTA and local infiltration anesthesia at the incision site should be jointly implemented.

### 4.1 Findings based on the study results

1. Pairwise comparisons of HR and MAP average values between the three groups revealed no statistically significant difference at T0 (before anesthetics administration) and T1 (the time of intubating or inserting laryngeal mask airway) (*p* > 0.05). All the three groups received preventive analgesia and dexmedetomidine for sedation and analgesia before surgery. Consequently, HR and BP decreased significantly, and intubation responses were well inhibited in all the three groups. Based on pairwise comparisons of HR and MAP average values between the three groups, differences between groups III and I and between groups III and II revealed a statistically significant difference at T2 (the time of incising skin), T3 (the time of disconnecting of the right sympathetic nerve), T4 (the time of disconnecting of the left sympathetic nerve), T5 (the time of withdrawing tracheal tube or laryngeal mask airway), and T6 (the time of transferring a patient to the PACU) (*p* < 0.05). The results revealed that laryngeal mask airways lead to less systemic stress responses than tracheal intubation. Laryngeal mask airways combined with local infiltration anesthesia at the incision site can ensure more comprehensive intraoperative and postoperative pain control. Therefore, group I had more stable BP and HR.
2. In this study, we examined the correlation between remifentanil and propofol and HR and MAP. The results revealed that when compared to group III, groups I and II used significantly smaller amount of remifentanil and propofol. This suggests that the preoperative use of local infiltrative anesthesia at the incision site can achieve a good preventive analgesic effect and reduce the dose of general anesthesia medications. The results revealed that when compared to group II, group I used a significantly smaller dose of remifentanil and propofol. This indicates that laryngeal mask airways cause less stimulation to patients during surgery and at extubation after surgery when compared to tracheal intubation, followed by a lower dose of anesthetics.
3. No patients in the observational group experienced throat discomfort after surgery, while patients in the control groups, namely groups II and III, experienced throat discomfort to various degrees postsurgery.
4. Groups I and II had lower VAS scores than Group III, with Group I scoring lower than Group II. This suggests that preoperative use of local infiltrative anesthesia at the incision site can achieve both an effective preventative analgesic effect and a comprehensive postoperative analgesic effect.

This study has several limitations—It has a small sample size and did not compare other anesthesia groupings such as general anesthesia without tracheal intubation and a combination of general anesthesia without tracheal intubation and local filtration anesthesia. This study requires further validation by conducting additional trials to provide a more reliable clinical treatment reference.

## 5 CONCLUSION

The combination of UFTA and local infiltration anesthesia during ETS results in fewer trauma and has an effective anesthetic impact. It is a safe and effective anesthesia method that adheres to the ERAS concept of fast‐track surgery and is worthy of promotion in clinical practice.

## AUTHOR CONTRIBUTIONS

Wenqing Zhang and Jian Lin conceived the idea and conceptualized the study. Wenqing Zhang, Jinglian Lin and Xuhang Chen collected the data. Xuhang Chen, Jinglian Lin and Huijuan Zhao analyzed the data. Huijuan Zhao and Wenqing Zhang drafted the manuscript, then Jian Lin and Zhijian Lin reviewed the manuscript. All authors read and approved the final draft.

## FUNDING INFORMATION

No external funding received to conduct this study.

## CONFLICT OF INTEREST STATEMENT

All authors declare that they have no competing interests.

## ETHICS STATEMENT

I confirm that I have read the Editorial Policy pages. This study was conducted with approval from the Ethics Committee of Zhangzhou Affiliated Hospital of Fujian medical University(Approval number 2023LWB224). This study was conducted in accordance with the declaration of Helsinki. Written informed consent was obtained from all participants.

## Supporting information


Figure S1.

## Data Availability

The data used to support the findings of this study are available from the corresponding author upon request.
